# Perfluoroalkyl substances influence DNA methylation in school-age children highly exposed through drinking water contaminated from firefighting foam: a cohort study in Ronneby, Sweden

**DOI:** 10.1093/eep/dvac004

**Published:** 2022-02-04

**Authors:** Yiyi Xu, Christian H Lindh, Tony Fletcher, Kristina Jakobsson, Karin Engström

**Affiliations:** School of Public Health and Community Medicine, Institute of Medicine, University of Gothenburg, Medicinaregatan 18A, Gothenburg 413 90, Sweden; Department of Laboratory Medicine, Division of Occupational and Environmental Medicine, Lund University, Scheelevägen 2, Lund 223 63, Sweden; Department of Social and Environmental Health Research, London School of Hygiene & Tropical Medicine, Keppel St, London WC1E 7HT, UK; School of Public Health and Community Medicine, Institute of Medicine, University of Gothenburg, Medicinaregatan 18A, Gothenburg 413 90, Sweden; Occupational and Environmental Medicine, Sahlgrenska University Hospital, Medicinaregatan 16 A, Gothenburg 413 90, Sweden; Department of Laboratory Medicine, EPI@LUND, Division of Occupational and Environmental Medicine, Lund University, Tornblad Institute, Biskopsgatan 9, Lund 223 62, Sweden

**Keywords:** epigenetics, environmental pollutant, perfluoroalkyl substance, PFAS, epigenetic aging, EPIC chip

## Abstract

Perfluoroalkyl substances (PFASs) are widespread synthetic substances with various adverse health effects. A potential mechanism of toxicity for PFASs is via epigenetic changes, such as DNA methylation. Previous studies have evaluated associations between PFAS exposure and DNA methylation among newborns and adults. However, no study has evaluated how PFASs influence DNA methylation among children of school age. In this exploratory study with school-age children exposed to PFASs through drinking water highly contaminated from firefighting foams, we aimed to investigate whether exposure to PFASs was associated with alteration in DNA methylation and epigenetic age acceleration. Sixty-three children aged 7–11 years from the Ronneby Biomarker Cohort (Sweden) were included. The children were either controls with only background exposure (*n* = 32; perfluorooctane sulfonic acid: median 2.8 and range 1–5 ng/ml) or those exposed to very high levels of PFASs (*n* = 31; perfluorooctane sulfonic acid: median 295 and range 190–464 ng/ml). These two groups were matched on sex, age, and body mass index. Genome-wide methylation of whole-blood DNA was analyzed using the Infinium MethylationEPIC BeadChip kit. Epigenetic age acceleration was derived from the DNA methylation data. Twelve differentially methylated positions and seven differentially methylated regions were found when comparing the high-exposure group to the control group. There were no differences in epigenetic age acceleration between these two groups (*P* = 0.66). We found that PFAS exposure was associated with DNA methylation at specific genomic positions and regions in children at school age, which may indicate a possible mechanism for linking PFAS exposure to health effects.

## Introduction

The ubiquitous spread of per- and polyfluoroalkyl substances (PFASs), a group of persistent organic pollutants, in the environment has become a global contamination problem. Drinking water contamination of PFASs occurs not only from PFAS production facilities but also from military and civilian firefighting training facilities where large quantities of aqueous film–forming foams have been used [1–3]. Public concern about PFAS contamination has increased since PFAS exposure has been linked to adverse health effects in all age groups [4]. A growing body of research has indicated that PFASs are associated with several adverse effects in children, such as reduced immune response, dyslipidemia, later age of puberty onset, and decreased bone mineral density [5, 6].

For these different health outcomes, the modes of PFAS toxicity are still not fully known. One possible mechanism is through alteration in DNA methylation that influences gene expression, affecting phenotype and health. DNA methylation occurs predominantly on cytosine (C) followed by guanine (G) residues, referred to as CpG sites. DNA methylation has been proposed as a possible molecular mechanism underlying adverse health effects of various environmental pollutants [7]. DNA methylation has the additional advantage of being a sensitive intermediate effect biomarker since it can be detected prior to developing subclinical changes or pathologies [8].

DNA methylation has been studied at background PFAS exposure levels in newborns from mother–child cohorts [9–12] and adults [13, 14]. Studies at high-exposure levels are still few: one study in adults after high perfluorooctanoic acid (PFOA) exposure [15] and one in adult women from Ronneby with highly contaminated drinking water, dominated by perfluorooctane sulfonic acid (PFOS) and perfluorohexane sulfonic acid (PFHxS) [16]. No study has evaluated how PFASs influence epigenetics in children of school age. Given that age can influence the epigenetic response [17] and children are particularly susceptible to exposure to environmental toxicants [18], studies investigating PFAS-related DNA methylation in children are warranted.

Additionally, data from whole-genome DNA methylation arrays can be used to estimate the biological age based on a number of age-dependent CpG signatures [19, 20]. Accelerated aging, i.e. the difference between biological age and chronological age, has been associated with exposure to organochloride pesticides and polybrominated biphenyls [21, 22], as well as increased mortality [23] and impaired physical and cognitive functions [24] in adults. Although we did not find any association between accelerated aging and PFASs in our previous study on adult women [16], it is still not known if PFAS exposure is associated with epigenetic aging in children. Alterations in epigenetic aging have been reported in children after environmental exposure to other pollutants, such as indoor air pollutants and parental smoking [25].

We conducted an epigenome-wide analysis to examine the associations between PFAS exposure and DNA methylation in children at school age by contrasting a control group with only background exposure to a group with very high PFAS exposure. We also evaluated if PFAS exposure was associated with epigenetic age acceleration.

## Materials and Methods

### Study Population

The study participants were selected from the Ronneby Biomarker Cohort and its reference group. The Ronneby cohort and reference group details have been previously published [3, 26]. In short, the Ronneby Biomarker Cohort comprises 3297 individuals aged 0–92 years from Ronneby, Sweden, a municipality that had a drinking water supply highly contaminated by PFASs, dominated by PFOS and PFHxS, for decades until December 2013 in one of its two waterworks. The reference group comprised 226 individuals (age range 5–59 years) from Karlshamn, a nearby municipality with an uncontaminated municipal drinking water supply and similar geodemographic characteristics. The average serum PFAS levels in the Ronneby cohort were about 10 to 100 times higher than in the reference group for different PFAS compounds [3].

This study focused on school-age children from 7 to 11 years old. The selection was based on serum PFOS levels and intended to be two distinct groups with a large contrast in exposure: one exposure group with highly exposed children and one control group with only background exposure. Among the 3297 participants from the Ronneby Biomarker Cohort with high PFAS exposure, there were 175 children aged 7–11 years. Additionally, there were 69 children in the same age range in the reference group with background exposure. We first restricted the Ronneby children to those with PFOS levels above the median to ensure a large contrast on exposure levels to the control group. We then performed frequency matching on age, sex, and body mass index (BMI) to selected participants from Ronneby with above-median PFOS and participants from Karlshamn. The high-exposure group consisted of 32 children residing in Ronneby with a mean serum PFOS at 295 ng/ml (range 190–464 ng/ml), and the control group then consisted of 32 children from Karlshamn, with a mean serum PFOS at 3 ng/ml (range 1–5 ng/ml). Unfortunately, data on parental smoking status were not available, nor data on potential confounders such as socioeconomic status, pubertal status, and parental occupation. Prior to the analyses, one sample (from the high-exposure group) was removed due to a too low amount of DNA, resulting in 63 study participants.

The study was carried out in accordance with The Code of Ethics of the World Medical Association (Declaration of Helsinki). The guardians of the participants gave informed written consent for biomarker studies. The Regional Ethical Review Board approved the study in Lund, Sweden (approved on 22 April 2014; approval number 2014/4).

### Serum PFAS Concentration Analysis

Serum PFAS concentrations were analyzed as described by Xu *et al.* [3]. Briefly, serum samples were collected and stored at −80°C at a biobank in Lund (Sweden) until lab analysis. Total non-isomer-specific PFHxS, PFOS, and PFOA levels were analyzed using liquid chromatography–tandem mass spectrometry at the Division of Occupational and Environmental Medicine in Lund (Sweden). The laboratory is qualified for PFAS analysis as an HBM4EU laboratory and participates in a quality control program for PFAS analysis (University of Erlangen-Nuremberg, Germany).

### DNA Methylation Analysis

DNA was extracted from the whole blood using Chemagic Maxiprep (PerkinElmer, Rodgau, Germany). The blood samples were taken simultaneously as the serum samples for PFAS analysis. DNA quality was evaluated using a NanoDrop spectrophotometer (NanoDrop Products, Wilmington, DE), based on the 260/280 nm ratio (all samples had a ratio ∼1.8, which is generally accepted as “pure” for DNA). Next, 500 ng DNA was bisulfite-treated using the EZ DNA Methylation kit (Zymo Research, Irvine, CA). DNA samples were randomized for distribution in a 96-well analysis plate. The entire bisulfite-treated DNA samples were used for the DNA methylation analysis. Genome-wide DNA methylation was determined at SNP&Seq, Uppsala, Sweden, using the Infinium MethylationEPIC BeadChip kit (lllumina, San Diego, CA) to analyze approximately 850 000 specific CpG sites in the genome. All beadchips were from the same batch.

### DNA Methylation Preprocessing

The statistical software R was used for the analysis of DNA methylation data. ChAMP [27, 28] and minfi (using ChAMP.load) [29, 30] were used for image processing, quality control, filtering, and normalization. Normalization was performed with beta-mixture quantile (BMIQ) normalization [31]. CpGs with sinngle nucleotide polymorphisms (SNPs), non-CpG probes, and multi-hit probes were filtered out. All 63 samples performed well (the detection *P*-value was below 0.01 for at least  >98% of CpGs for all samples). CpGs for which the detection *P*-value was above 0.01 in more than 20% of the samples were removed. Probes in the X or Y chromosome were removed in the analyses including all individuals. 740 630 CpGs were retained in the analyses including all individuals, and 757 346 CpGs were retained for the sex-stratified analyses.

DNA methylation data were used to determine the proportion of different cell types (B cells, CD4^+^ T cells, CD8^+^ T cells, granulocytes, monocytes, and natural killer cells) from reference datasets for sorted samples using the function estimateCellCounts in minfi [30] as described by Houseman *et al.* [32]. These proportions were later evaluated as potential covariates in the statistical models. Singular value decomposition (SVD) [33] was performed in ChAMP to identify the technical and biological variables associated with DNA methylation. The SVD analyses showed that the technical variable “slide” (i.e. physical position in the analysis plate) accounted for a substantial fraction of variation (*P* < 0.05) in principal component 2; thus, “slide” was then included in ComBat adjustments [34, 35] using ChAMP.

### Predicted Epigenetic Age

The biological age was estimated using the epigenetic “skin and blood clock,” a highly robust DNA methylation age estimator based on 391 CpGs [20]. The individual epigenetic age acceleration was then estimated as the residual by regressing the estimated biological age to the chronological age. A positive value of age acceleration indicates a faster aging than expected, i.e. an accelerating epigenetic clock.

### Statistical Analysis

The SVD analyses that identified variables associated with DNA methylation showed that estimated fractions of B cells, CD4^+^ T cells, CD8^+^ T cells, and neutrophils had a *P*-value <0.05 in the first two principal components. Due to the strong correlation between the fractions of the different cell types, the model described below was only adjusted for neutrophil fraction, which was the cell type that showed the strongest association with DNA methylation (data not shown).

Differentially methylated positions (DMPs) were evaluated by fitting a linear regression model to each CpG using the R package limma [36], with adjustments for the estimated fraction of neutrophils. *M*-values (the log2 ratios of the intensities of methylated probe versus unmethylated probe) were used as the dependent variable [37]. Empirical Bayes smoothing was applied to the standard errors with a robust selection against outlier sample variances. Furthermore, *P*-values were adjusted for multiple comparisons for all CpGs by the Benjamini–Hochberg false discovery rate (FDR) method to obtain *q*-values. CpGs with a *q*-value of 0.05 or lower were considered being DMPs. Analyses were performed for 1) all individuals and 2) stratified for sex.

Differentially methylated regions (DMRs) were evaluated using the R package DMRCate [38]. Gaussian kernel bandwidth for smoothed-function estimation was set to 1000 nucleotides, and the scaling factor for bandwidth was set to 2. DMRs that were at least two CpGs long were included. DMRs are ranked by Fisher’s multiple comparison statistics. Regions with a *q*-value of 0.05 or lower were considered being DMRs.

The correlation between biological age and chronological age was evaluated using Spearman correlation. The difference in epigenetic age acceleration between the high-exposure and control groups was analyzed using the Mann–Whitney U test. A *P*-value of 0.05 or lower was considered statistically significant.

### Gene Ontology and KEGG Analyses

Gene ontology (GO) and Kyoto Encyclopedia of Genes and Genomes (KEGG) analyses [GO/KEGG accessed May 2021] [39–41] were run for genes that had CpGs with a *q*-value <0.1 in the DMP analyses. GO and KEGG analyses were performed using the gometh function in the R package missMethyl, which considers the differing number of probes per gene present on the array.

## Results

Table 1 shows the characteristics and serum levels of PFASs of the 63 children in this study. Even though we selected study participants based on their PFOS level, there were large contrasts between the high-exposure group and the control group in serum levels of PFOA and PFHxS as well.

**Table 1:** Descriptive statistics [count (%) or median (range)] in the control group and the high-exposure group

Variable	Control group (*n* = 32)	High-exposure group (*n* = 31)
Sex (% girls)	16 (50)	16 (52)
Age (years)	9 (6–11)	9 (6–11)
BMI (kg/m^2^)	17 (15–21)	17 (14–23)
PFOS (ng/ml)	2.8 (1.0–4.7)	295 (190–464)
PFHxS (ng/ml)	0.5 (0.3–1.6)	277 (147–410)
PFOA (ng/ml)	1.4 (0.7–3.5)	19 (12–32)

### Epigenome-wide Analysis of DMPs and DMRs

We first evaluated the DMPs among all study participants. Comparing the high-exposure and control groups, we identified 12 DMPs (*q* < 0.05, Table 2), and there were 364 CpGs with a *q*-value <0.1 (Supplemental material, Excel Supplemental Table S1). Most of the 12 DMPs (75%) were hypermethylated in the high-exposure group compared to the control group (Table 2).

**Table 2:** The 12 DMPs (*q* < 0.05) between the high-exposure group and control group

CpG	Chr	Position^a^	Gene	Gene name	2^^^logFC (95% CI)^b^	*q*-Value
cg15732078	10	86097061	*CCSER2*	Coiled-coil serine-rich protein 2	1.36 (1.23, 1.49)	0.025
cg16593554	18	74813401	*MBP*	Myelin basic protein	1.47 (1.30, 1.67)	0.025
cg02556924	15	101661693	NA^c^		0.78 (0.72, 0.85)	0.033
cg01899620	13	113689422	*MCF2L*	MCF.2 cell line derived transforming sequence like	0.77 (0.71, 0.85)	0.034
cg23696710	11	17260094	NA		1.42 (1.27, 1.61)	0.034
cg05371502	8	67522986	*MYBL1*	MYB proto-oncogene like 1	1.30 (1.18, 1.42)	0.046
cg25840780	20	50156843	*NFATC2*	Nuclear factor of activated T cells 2	1.42 (1.25, 1.61)	0.046
cg24295885	4	57267050	*PPAT*	Phosphoribosyl pyrophosphate amidotransferase	1.31 (1.19, 1.45)	0.046
cg02051878	15	74502715	*STRA6*	Stimulated by retinoic acid 6	1.28 (1.17, 1.40)	0.046
cg07503415	6	44171877	NA		1.26 (1.16, 1.36)	0.046
cg13562278	3	31207280	NA		1.32 (1.19, 1.45)	0.046
cg23821954	18	46497074	NA		0.74 (0.66, 0.82)	0.046

Abbreviations: Chr, chromosome; CI, confidence interval; *q*-value, FDR-adjusted *P*-value using the Benjamini–Hochberg method; logFC, binary logarithmic fold change.

a Chromosomal coordinates of the CpG (Build 37).

b 2^^^logFC, two to the power of logFC. LogFC denotes *β*_1_ from the following robust regression model: *M*-value = *β*_1_* *× group comparison + *β*_2_* *× estimated fraction of neutrophils. The control group is the reference group.

c NA, not annotated, i.e. the CpG is not present in any known gene.

We then performed a DMP analysis in each sex stratum, but none of the CpGs had a *q*-value <1.0. Top CpG for girls was cg14111928 in lysine acetyltransferase 6B (*MYST1*) [2^^^log fold change (FC) = 1.24, 95% confidence interval (CI): 1.14–1.31, unadjusted *P*-value = 0.00001, *q* = 1.0], while top CpG for boys was cg16813892 in metal response element-binding transcription factor 2 (*MTF2*) (2^^^logFC = 1.43, 95% CI: 1.26–1.64, unadjusted *P*-value = 0.000002, *q* = 1.0). Top 10 CpGs of the sex-stratified analysis are presented in Supplemental material, Excel Supplemental Tables S2 and S3.

We then evaluated DMRs. We found seven DMRs (Fisher’s multiple comparison statistic *q* < 0.05) (Table 3) in the high-exposure group compared to the control group. To note, the top DMP cg15732078 in coiled-coil serine-rich protein 2 (*CCSER2)* was also part of one top DMR, although this was a small DMR encompassing only two CpGs. DMR analyses were also performed in each sex stratum, but no regions showed a *q*-value <0.05.

**Table 3:** The seven DMRs (*q* < 0.05) between the high-exposure group and control group

Gene(s)	Gene names	Number of CpGs	Location (Chr: start, end)	Mean 2^^^logFC within the region^a^	*q*-Value
*LPCAT1*	Lysophosphatidylcholine acyltransferase 1	5	chr5: 1503259-1503979	1.022	0.022
*CCSER2*	Coiled-coil serine-rich protein 2	2	chr10: 86096789-86097061	1.024	0.029
*BST2, MVB12A*	Bone marrow stromal cell antigen 2, multivesicular body subunit 12A	13	chr19: 17515486-17517762	1.013	0.030
*RASAL2*	RAS protein activator like 2	2	chr1: 178244359-178245008	1.028	0.033
*TLDC2*	TBC/LysM-associated domain containing 2	8	chr20: 35503983-35504553	1.020	0.043
*MYO7A*	Myosin VIIA	3	chr6: 109035178-109036145	1.05	0.045
*ITPR1*	Inositol 1,4,5-trisphosphate receptor type 1	3	chr3: 4790361-4790790	1.03	0.050

Abbreviations: Chr: chromosome; *q*-value, FDR-adjusted *P*-value using the Benjamini–Hochberg method; logFC, binary logarithmic fold change.

a 2^^^logFC, two to the power of logFC. LogFC denotes *β*_1_ from the following robust regression model: *M*-value = *β*_1_* *× group comparison + *β*_2_* *× estimated fraction of neutrophils. The control group is the reference group.

^b^ Fisher’s multiple comparison statistic *q*-value.

### Gene Ontology/KEGG Analyses for DMPs

Next, we tested for potential enrichment of GO and KEGG terms for the CpGs with a *q*-value <0.1 annotated to genes. We found no significant terms after the correction for multiple testing (FDR < 0.1), and all terms had an FDR of 1.0. The top five KEGG and GO terms, ranked by *P*-value, are listed in Table 4. We did not perform any pathway analysis for the sex-stratified data since there was no CpG with a *q*-value <0.1 in the sex-stratified analyses.

**Table 4:** Top KEGG and GO terms enriched by the CpGs with a *q*-value <0.1, ranked by *P*-value

Term	Term ID	*P*-value^a^	Total number of genes in Term	Number of genes from CpGs with a *q*-value <0.1 in this study
Gene ontology				
Regulation of developmental process	GO:0050793	0.000168	2538	35
Hemopoiesis	GO:0030097	0.000328	839	16
Osteoclast development	GO:0036035	0.000386	17	3
Positive regulation of myoblast fusion	GO:1901741	0.000474	19	3
Hematopoietic or lymphoid organ development	GO:0048534	0.000619	881	16
KEGG				
Apoptosis	KEGG:04210	0.01	132	4.5
Basal transcription factors	KEGG:03022	0.02	39	2
Mineral absorption	KEGG:04978	0.04	53	2
Lysosome	KEGG:04142	0.04	123	3
Glycosphingolipid biosynthesis—globo and isoglobo series	KEGG:00603	0.08	13	1

a All terms had a *q*-value of 1.0.

### Epigenetic Age Acceleration

Lastly, we investigated whether PFAS exposure was associated with epigenetic age acceleration. There was a high correlation between the epigenetic age and chronological age in the study population (*r_s_* = 0.58, *P* < 0.001). We did not observe any difference in epigenetic age acceleration between the high-exposure and control groups (*P* = 0.82 from the Mann–Whitney test).

## Discussion

This is, to our knowledge, the first study to conduct an epigenome-wide analysis to examine the association between PFAS exposure and DNA methylation in a highly exposed group of children at school age. In this exploratory study with 63 children, 12 DMPs and 7 DMRs were significantly associated with high PFAS exposure. However, there was no association between PFAS exposure and epigenetic age acceleration. These observations suggest that PFAS exposure is associated with DNA methylation for some specific genes. However, future and larger studies are needed to confirm these associations and convey potential causality between PFAS exposure and DNA methylation and potentially adverse health outcomes.

Although there have been some studies evaluating PFASs and DNA methylation in newborns at background exposure levels [9–12], no studies involving children beyond the newborn stage have evaluated the influence of PFAS exposure on DNA methylation. Investigating PFAS-related DNA methylation in different age groups is essential, given that DNA methylation can change during the entire life span [42]. The most significant age-related dynamics of DNA methylation manifest in early childhood from 2 to 10 years old [43]. Thus, due to the difference in exposure levels between previous studies and ours and the age-related dynamics of DNA methylation during childhood, it is not known how comparable these findings are to studies in newborns at background exposure levels. Additionally, studies at high-exposure levels are few and restricted to adults. There were no overlaps of DMPs or DMRs with those in our previous study comprising women in the same cohort [16]. One reason for this could be aged-related differences in methylation.

In our study, *RASAL2* was found to be hypermethylated in the group with high PFAS exposure compared to controls, indicating possible epigenetic silencing. *RASAL2* gene encodes a protein (RASAL2) that belongs to a family of Ras guanosine triphophate (GTP)ase-activating proteins (RasGAPs), stimulating the GTPase activity of wild-type RAS [44]. This gene has been identified as a functional tumor suppressor [45], and downregulation of the RASAL2 protein has been reported in various cancers, e.g. lung, breast, bladder, and colorectal cancer [45–48]. *RASAL2* hypermethylation has been associated with higher stages and grades and worse survival rates in patients with renal cell carcinoma [49]. Interestingly, a recently published case-control study observed a higher risk of renal cell carcinoma associated with PFOA exposure [50], suggesting that PFOA is a renal carcinogen [51]. Our research group found modestly increased risks of kidney cancer in the Ronneby population with high PFAS exposure through drinking water [52]. Kingsley *et al.* found that RAS p21 protein activator 3 (*RASA3)*, another gene coding RasGAPs, was hypomethylated with increased maternal exposure to PFOA in cord blood samples in 44 mother–infant pairs with background exposure levels [9]. Although *RASAL2* and *RASA3* encode RasGAPs, they may have different biological functions; thus, a direct comparison to our findings is difficult. Future studies with a larger sample size are needed to verify our findings of hypermethylated *RASAL2* after being highly exposed to PFASs.

*ITPR1*, a gene encoding an intracellular receptor for inositol 1,4,5-trisphosphate and mediating calcium release from the endoplasmic reticulum, was also found to be hypermethylated in the high PFAS exposure group compared to the control group. This gene has not been reported in other human studies investigating PFAS-associated epigenetic changes. However, in an animal study with PFOS-exposed rats, alteration in miRNAs encoding *ITPR1* was found in neonatal rats’ brain samples, indicating that PFOS may affect calcium actions through interfering with *ITPR1* and cause adverse effects on the nervous system [53].

The other genes encompassing DMPs or DMRs in our study have not been previously associated with PFAS exposure, while some of them may be of interest considering their biological function. MCF.2 cell line derived transforming sequence like (*MCF2L*), a gene encoding a guanine nucleotide exchange factor that interacts specifically with the GTP-bound Rac1 and plays a role in the Rho/Rac signaling pathways [54], was found to be hypomethylated in the high PFAS exposure group compared to the control group, indicating that PFAS exposure may inhibit MCF2L expression. Given that MCF2L was less expressed in osteoporosis patients than controls in one study [55] and PFASs have been associated with lower bone mineral density in mid-childhood [5], an association linking PFAS exposure, MCF2L expression, and bone health warrants further studies. In addition, osteoclast development was one GO term related to PFAS exposure, although it was not significant after FDR adjustments.

In the KEGG pathway analysis, apoptosis was the suggested pathway with the smallest *P*-value, although the significance disappeared after FDR correction. In animal and cell studies, PFASs were shown to induce apoptosis in cells through the production of reactive oxygen species and oxidative stress [56, 57] and the modulation of gene regulation via peroxisome proliferator-activated receptors (PPARs) and nuclear factor kappa beta (NF-κB) transcription [4, 58]. Our previous study on Ronneby women found PPARα/retinoid X receptor alpha (RXRα) activation as an enriched pathway [16]. However, the interpretation should be cautious due to the lack of statistical significance.

We did not note any association between PFAS exposure and epigenetic age acceleration among these school-age children. In highly PFAS-exposed women aged 20–45 years from the Ronneby Biomarker Cohort, no association with epigenetic age acceleration was seen either [16]. The main limitation of this study is the sample size which reduced the statistical power. Since the PFAS is an endocrine disruptor, sex differences in PFAS-related DNA methylation are plausible, but this study did not have enough power to elucidate sex-specific effects. Another limitation is the lack of information about socioeconomic status, pubertal status, parental occupation of the children, etc., which may be potential confounders. However, the control and highly exposed PFAS groups came from two closely situated municipalities with similar geodemographic statuses. Given the small sample size, we used frequency matching for age, sex, and BMI to control pubertal status. A strength of the study is the large contrast in PFAS exposure, comparing children with a general population background exposure to children with very high exposures, especially for PFOS and PFHxS.

Our study among children indicates that PFAS exposure is associated with DNA methylation changes at specific sites. Further studies with larger sample sizes are needed to validate the findings and couple the PFAS-associated changes in DNA methylation to phenotypical effects of PFASs.

In conclusion, we found that PFAS exposure was associated with DNA methylation at specific genomic positions and regions in children at school age. Most DMPs and DMRs were hypermethylated in the high-exposure group compared to the control group.

## Supplementary Material

dvac004_Supp

## Data Availability

Data are available at 10.5061/dryad.kwh70rz5k.

## References

[1] Barzen-Hanson KA , Roberts SC, Choyke S et al. Discovery of 40 classes of per-and polyfluoroalkyl substances in historical aqueous film-forming foams (AFFFs) and AFFF-impacted groundwater. *Environ**Sci Technol* 2017;51:2047–57. 10.1021/acs.est.6b05843 28098989

[2] Guelfo JL , Adamson DT. Evaluation of a national data set for insights into sources, composition, and concentrations of per-and polyfluoroalkyl substances (PFASs) in US drinking water. *Environ Pollut* 2018;236:505–13. 29427949 10.1016/j.envpol.2018.01.066 PMC5849529

[3] Xu Y , Nielsen C, Li Y et al. Serum perfluoroalkyl substances in residents following long-term drinking water contamination from firefighting foam in Ronneby, Sweden. *Environ Int* 2021;147:106333. 10.1016/j.envint.2020.106333 33360412

[4] European Food Safety Authority . Risk to human health related to the presence of perfluorooctane sulfonic acid and perfluorooctanoic acid in food. *EFSA J* 2018;16:e05194. 10.2903/j.efsa.2018.5194 PMC7009575 32625773

[5] Cluett R , Seshasayee SM, Rokoff LB et al. Per- and polyfluoroalkyl substance plasma concentrations and bone mineral density in midchildhood: a cross-sectional study (Project Viva, United States). *Environ Health Perspect* 2019;127:87006. 10.1289/EHP4918 PMC6792359 31433236

[6] Rappazzo KM , Coffman E, Hines EP. Exposure to perfluorinated alkyl substances and health outcomes in children: a systematic review of the epidemiologic literature. *Int J Environ Res Public Health* 2017;14:691.doi: 10.3390/ijerph14070691. PMC5551129 28654008

[7] Baccarelli A , Bollati V. Epigenetics and environmental chemicals. *Curr Opin Pediatr* 2009;21:243–51. 19663042 10.1097/mop.0b013e32832925cc PMC3035853

[8] Maunakea AK , Chepelev I, Zhao K. Epigenome mapping in normal and disease states. *Circ Res* 2010;107:327–39. 20689072 10.1161/CIRCRESAHA.110.222463 PMC2917837

[9] Kingsley SL , Kelsey KT, Butler R et al. Maternal serum PFOA concentration and DNA methylation in cord blood: a pilot study. *Environ**Res* 2017;158:174–8. 10.1016/j.envres.2017.06.013 PMC5554452 28645023

[10] Kobayashi S , Azumi K, Goudarzi H et al. Effects of prenatal perfluoroalkyl acid exposure on cord blood IGF2/H19 methylation and ponderal index: the Hokkaido study. *J Expo Sci Environ Epidemiol* 2017;27:251–9. 27553991 10.1038/jes.2016.50

[11] Miura R , Araki A, Miyashita C et al. An epigenome-wide study of cord blood DNA methylations in relation to prenatal perfluoroalkyl substance exposure: the Hokkaido study. *Environ**Int* 2018;115:21–8. 10.1016/j.envint.2018.03.004 29544137

[12] Starling AP , Liu C, Shen G et al. Prenatal exposure to per- and polyfluoroalkyl substances, umbilical cord blood DNA methylation, and cardio-metabolic indicators in newborns: The Healthy Start Study. *Environ Health Perspect* 2020;128:127014. 10.1289/EHP6888 PMC7759236 33356526

[13] Leter G , Consales C, Eleuteri P et al. Exposure to perfluoroalkyl substances and sperm DNA global methylation in Arctic and European populations. *Environ**Mol Mutagen* 2014;55:591–600. 10.1002/em.21874 24889506

[14] van den Dungen MW , Murk AJ, Kampman E et al. Association between DNA methylation profiles in leukocytes and serum levels of persistent organic pollutants in Dutch men. *Environ**Epigenet* 2017;3:dvx001.doi: 10.1093/eep/dvx001. PMC5804541 29492303

[15] Watkins DJ , Wellenius GA, Butler RA et al. Associations between serum perfluoroalkyl acids and LINE-1 DNA methylation. *Environ**Int* 2014;63:71–6. 10.1016/j.envint.2013.10.018 PMC4181536 24263140

[16] Xu Y , Jurkovic-Mlakar S, Lindh CH et al. Associations between serum concentrations of perfluoroalkyl substances and DNA methylation in women exposed through drinking water: a pilot study in Ronneby, Sweden. *Environ Int* 2020;145:106148. 10.1016/j.envint.2020.106148 33007577

[17] Day K , Waite LL, Thalacker-Mercer A et al. Differential DNA methylation with age displays both common and dynamic features across human tissues that are influenced by CpG landscape. *Genome Biol* 2013;14:R102. 10.1186/gb-2013-14-9-r102 PMC4053985 24034465

[18] Faustman EM , Silbernagel SM, Fenske RA et al. Mechanisms underlying children’s susceptibility to environmental toxicants. *Environ Health Perspect* 2000;108:13–21. 10.1289/ehp.00108s113 PMC1637781 10698720

[19] Horvath S . DNA methylation age of human tissues and cell types. *Genome Biol* 2013;14:3156. 10.1186/gb-2013-14-10-r115 PMC4015143 24138928

[20] Horvath S , Oshima J, Martin GM et al. Epigenetic clock for skin and blood cells applied to Hutchinson Gilford Progeria Syndrome and ex vivo studies. *Aging* 2018;10:1758. 10.18632/aging.101508 PMC6075434 30048243

[21] Curtis SW , Cobb DO, Kilaru V et al. Environmental exposure to polybrominated biphenyl (PBB) associates with an increased rate of biological aging. *Aging* 2019;11:5498. 10.18632/aging.102134 PMC6710070 31375641

[22] Lind PM , Salihovic S, Lind L. High plasma organochlorine pesticide levels are related to increased biological age as calculated by DNA methylation analysis. *Environ**Int* 2018;113:109–13. 10.1016/j.envint.2018.01.019 29421399

[23] Marioni RE , Shah S, McRae AF et al. DNA methylation age of blood predicts all-cause mortality in later life. *Genome Biol* 2015;16:25. 10.1186/s13059-015-0584-6 PMC4350614 25633388

[24] Marioni RE , Shah S, McRae AF et al. The epigenetic clock is correlated with physical and cognitive fitness in the Lothian Birth Cohort 1936. *Int J Epidemiol* 2015;44:1388–96. 25617346 10.1093/ije/dyu277 PMC4588858

[25] de Prado-bert P , Ruiz-Arenas C, Vives-Usano M et al. The early-life exposome and epigenetic age acceleration in children. *Environ Int* 2021;155:106683. 10.1016/j.envint.2021.106683 34144479

[26] Xu Y , Jurkovic-Mlakar S, Li Y et al. Association between serum concentrations of perfluoroalkyl substances (PFAS) and expression of serum microRNAs in a cohort highly exposed to PFAS from drinking water. *Environ**Int* 2020;136:105446. 10.1016/j.envint.2019.105446 31926437

[27] Morris TJ , Butcher LM, Feber A et al. ChAMP: 450k chip analysis methylation pipeline. *Bioinformatics* 2014;30:428–30. 24336642 10.1093/bioinformatics/btt684 PMC3904520

[28] Tian Y , Morris TJ, Webster AP et al. ChAMP: updated methylation analysis pipeline for Illumina BeadChips. *Bioinformatics* 2017;33:3982–4. 28961746 10.1093/bioinformatics/btx513 PMC5860089

[29] Aryee MJ , Jaffe AE, Corrada-Bravo H et al. Minfi: a flexible and comprehensive Bioconductor package for the analysis of Infinium DNA methylation microarrays. *Bioinformatics* 2014;30:1363–9. 24478339 10.1093/bioinformatics/btu049 PMC4016708

[30] Fortin J-P , Triche JTJ, Hansen KD. %J B. Preprocessing, normalization and integration of the Illumina HumanMethylationEPIC array with minfi. *Bioinformatics* 2017;33:558–60. 28035024 10.1093/bioinformatics/btw691 PMC5408810

[31] Teschendorff AE , Marabita F, Lechner M et al. A beta-mixture quantile normalization method for correcting probe design bias in Illumina Infinium 450 k DNA methylation data. *Bioinformatics* 2013;29:189–96. 23175756 10.1093/bioinformatics/bts680 PMC3546795

[32] Houseman EA , Accomando WP, Koestler DC et al. DNA methylation arrays as surrogate measures of cell mixture distribution. *BMC Bioinform* 2012;13:86. 10.1186/1471-2105-13-86 PMC3532182 22568884

[33] Teschendorff AE , Menon U, Gentry-Maharaj A et al. An epigenetic signature in peripheral blood predicts active ovarian cancer. *PLoS One* 2009;4:e8274. 10.1371/journal.pone.0008274 PMC2793425 20019873

[34] Johnson WE , Li C, Rabinovic A. Adjusting batch effects in microarray expression data using empirical Bayes methods. *Biostat Oxf Engl* 2007;8:118–27. 10.1093/biostatistics/kxj037 16632515

[35] Leek JT , Johnson WE, Parker HS et al. sva: Surrogate variable analysis R package version 3.10. 0. 2014;10:B9.

[36] Ritchie ME , Phipson B, Wu D et al. Limma powers differential expression analyses for RNA-sequencing and microarray studies. *Nucleic Acids Res* 2015;43:e47. 10.1093/nar/gkv007 PMC4402510 25605792

[37] Irizarry RA , Ladd-Acosta C, Carvalho B et al. Comprehensive high-throughput arrays for relative methylation (CHARM). *Genome Res* 2008;18:780–90. 18316654 10.1101/gr.7301508 PMC2336799

[38] Peters A , Hampel R, Cyrys J et al. Elevated particle number concentrations induce immediate changes in heart rate variability: a panel study in individuals with impaired glucose metabolism or diabetes. *Part Fibre Toxicol* 2015;12:7. 10.1186/s12989-015-0083-7 PMC4379544 25888845

[39] Kanehisa M , Goto S. KEGG: Kyoto encyclopedia of genes and genomes. *Nucleic Acids Res* 2000;28:27–30. 10592173 10.1093/nar/28.1.27 PMC102409

[40] Kanehisa M , Sato Y, Furumichi M et al. New approach for understanding genome variations in KEGG. *Nucleic Acids Res* 2019;47:D590–5. 30321428 10.1093/nar/gky962 PMC6324070

[41] Kanehisa M , Sato Y. KEGG mapper for inferring cellular functions from protein sequences. *Protein Sci* 2020;29:28–35. 31423653 10.1002/pro.3711 PMC6933857

[42] Bjornsson HT , Sigurdsson MI, Fallin MD et al. Intra-individual change over time in DNA methylation with familial clustering. *JAMA* 2008;299:2877–83. 18577732 10.1001/jama.299.24.2877 PMC2581898

[43] Gervin K , Andreassen BK, Hjorthaug HS et al. Intra-individual changes in DNA methylation not mediated by cell-type composition are correlated with aging during childhood. *Clin Epigenetics* 2016;8:110. 10.1186/s13148-016-0277-3 PMC5073885 27785156

[44] King PD , Lubeck BA, Lapinski PE. Nonredundant functions for Ras GTPase-activating proteins in tissue homeostasis. *Sci Signal* 2013;6:re1. 10.1126/scisignal.2003669 PMC5483993 23443682

[45] McLaughlin SK , Olsen SN, Dake B et al. The RasGAP gene, RASAL2, is a tumor and metastasis suppressor. *Cancer Cell* 2013;24:365–78. 24029233 10.1016/j.ccr.2013.08.004 PMC3822334

[46] Hui K , Gao Y, Huang J et al. RASAL2, a RAS GTPase-activating protein, inhibits stemness and epithelial-mesenchymal transition via MAPK/SOX2 pathway in bladder cancer. *Cell Death Dis* 2017;8:e2600. 10.1038/cddis.2017.9 PMC5386500 28182001

[47] Jia Z , Liu W, Gong L et al. Downregulation of RASAL2 promotes the proliferation, epithelial-mesenchymal transition and metastasis of colorectal cancer cells. *Oncol Lett* 2017;13:1379–85. 28454265 10.3892/ol.2017.5581 PMC5403410

[48] Li N , Li S. RASAL2 promotes lung cancer metastasis through epithelial-mesenchymal transition. *Biochem Biophys Res Commun* 2014;455:358–62. 25446096 10.1016/j.bbrc.2014.11.020

[49] Hui K , Yue Y, Wu S et al. The expression and function of RASAL2 in renal cell carcinoma angiogenesis. *Cell Death Dis* 2018;9:881. 10.1038/s41419-018-0898-x PMC6115459 30158581

[50] Shearer JJ , Callahan CL, Calafat AM et al. Serum concentrations of per- and polyfluoroalkyl substances and risk of renal cell carcinoma. *J Natl Cancer Inst* 2021;113:580–7. 32944748 10.1093/jnci/djaa143 PMC8096365

[51] Steenland K , Winquist A. PFAS and cancer, a scoping review of the epidemiologic evidence. *Environ Res* 2021;194:110690. 10.1016/j.envres.2020.110690 PMC7946751 33385391

[52] Li H , Hammarstrand S, Midberg B et al. Cancer incidence in a Swedish cohort with high exposure to perfluoroalkyl substances in drinking water. *Environ Res* 2022;204:112217. 10.1016/j.envres.2021.112217 34662573

[53] Wang F , Liu W, Ma J et al. Prenatal and neonatal exposure to perfluorooctane sulfonic acid results in changes in miRNA expression profiles and synapse associated proteins in developing rat brains. *Environ Sci Technol* 2012;46:6822–9. 22594572 10.1021/es3008547

[54] Zhang YJ , Wen CL, Qin YX et al. Establishment of a human primary pancreatic cancer mouse model to examine and investigate gemcitabine resistance. *Oncol Rep* 2017;38:3335–46. 29039610 10.3892/or.2017.6026 PMC5783578

[55] Mao JH , Sui YX, Ao S et al. miR-140-3p exhibits repressive functions on preosteoblast viability and differentiation by downregulating MCF2L in osteoporosis. *Vitro Cell Dev Biol - Anim* 2020;56:49–58. 10.1007/s11626-019-00405-9 31732956

[56] Kim HS , Jun Kwack S, Sik Han E et al. Induction of apoptosis and CYP4A1 expression in Sprague-Dawley rats exposed to low doses of perfluorooctane sulfonate. *J Toxicol Sci* 2011;36:201–10. 21467747 10.2131/jts.36.201

[57] Zhang L , Krishnan P, Ehresman DJ et al. Editor’s highlight: perfluorooctane sulfonate-choline ion pair formation: a potential mechanism modulating hepatic steatosis and oxidative stress in mice. *Toxicol Sci* 2016;153:186–97. 27413108 10.1093/toxsci/kfw120

[58] DeWitt JC , Peden-Adams MM, Keller JM et al. Immunotoxicity of perfluorinated compounds: recent developments. *Toxicol Pathol* 2012;40:300–11. 22109712 10.1177/0192623311428473

