# Supplementary material for: Perfluoroalkyl substances influence DNA methylation in school-age children highly exposed through drinking water contaminated from firefighting foam: a cohort study in Ronneby, Sweden
Source: Environ Epigenet. 2022 Feb 4;8(1):dvac004. doi: 10.1093/eep/dvac004 (PMC8931254; doi:10.1093/eep/dvac004)
Supplement: dvac004_Supp [file dvac004_supp.zip › Supplemental xuetal.pdf]

Supplemental Table S1. DMPs with q below 0.1.

CpG site	2^logFC	CI left	CI right	q. Value	CHR	Position	Gene
cg15732078	1,36	1,23	1,49	0.025	10	86097061	*CCSER2*
cg16593554	1,47	1,30	1,67	0.025	18	74813401	*MBP*
cg02556924	0,78	0,72	0,85	0.033	15	101661693	
cg01899620	0,77	0,71	0,85	0.034	13	113689422	*MCF2L*
cg23696710	1,42	1,27	1,61	0.034	11	17260094	
cg05371502	1,30	1,18	1,42	0.046	8	67522986	*MYBL1*
cg25840780	1,42	1,25	1,61	0.046	20	50156843	*NFATC2*
cg24295885	1,31	1,19	1,45	0.046	4	57267050	*PPAT*
cg02051878	1,28	1,17	1,40	0.046	15	74502715	*STRA6*
cg07503415	1,26	1,16	1,36	0.046	6	44171877	
cg13562278	1,32	1,19	1,45	0.046	3	31207280	
cg23821954	0,74	0,66	0,82	0.046	18	46497074	
cg19056691	1,29	1,17	1,42	0.053	12	111777251	*CUX2*
cg04961615	1,52	1,29	1,78	0.053	6	137064665	*MAP3K5*
cg06760899	1,21	1,13	1,29	0.053	22	27069320	*MIAT*
cg04501557	1,43	1,25	1,64	0.053	2	223572638	*MOGAT1*
cg00524374	1,29	1,17	1,42	0.053	10	3894968	
cg00591068	1,57	1,32	1,87	0.053	15	89623859	
cg02295919	1,34	1,20	1,48	0.053	20	56493122	
cg02473484	1,48	1,27	1,73	0.053	20	52371088	
cg08461876	1,88	1,48	2,38	0.053	1	59437584	
cg08505065	1,33	1,20	1,48	0.053	9	4350277	
cg12806639	1,28	1,17	1,41	0.053	11	67917666	
cg16238920	0,83	0,78	0,89	0.053	8	129281825	
cg00100420	1,36	1,21	1,53	0.054	4	106009831	
cg21579666	1,23	1,13	1,33	0.06	17	75306740	*SEPT9*
cg16820973	1,39	1,22	1,57	0.061	12	32711698	*FGD4*
cg02592510	1,45	1,26	1,68	0.061	12	131605571	*GPR133*
cg09028747	1,38	1,21	1,56	0.061	2	42374937	*LOC102723824*
cg12699728	1,22	1,13	1,32	0.061	15	45461636	*SHF*
cg01841520	1,21	1,13	1,29	0.061	2	43238433	
cg03149616	1,39	1,22	1,59	0.061	8	19759158	
cg03429223	1,49	1,27	1,74	0.061	1	110438516	
cg11374718	1,58	1,32	1,88	0.063	6	29273773	*OR14J1*
cg25573644	1,19	1,11	1,27	0.063	1	178245008	*RASAL2*
cg23017141	0,80	0,73	0,88	0.068	6	24887369	*FAM65B*
cg22754866	1,32	1,18	1,47	0.068	10	99771121	*CRTAC1*
cg21068078	1,29	1,16	1,42	0.068	18	13488504	*LDLRAD4*
cg23803838	1,38	1,21	1,56	0.07	12	39064923	*CPNE8*
cg12807206	1,46	1,26	1,69	0.07	2	157324457	*GPD2*
cg07897699	1,33	1,19	1,48	0.07	3	4790491	*ITPR1*
cg12654620	1,31	1,18	1,46	0.07	8	90741449	*LOC101929709*
cg08450501	1,32	1,18	1,47	0.07	3	136580965	*NCK1*
cg05696848	1,27	1,15	1,39	0.07	16	27257330	*NSMCE1*
cg16436566	1,21	1,13	1,32	0.07	16	67879671	*NUTF2*
cg05005367	0,72	0,63	0,82	0.07	10	112408153	*RBM20*
cg10608169	1,39	1,21	1,58	0.07	9	37964630	*SHB*
cg00774089	1,52	1,28	1,79	0.07	5	118676727	*TNFAIP8*

cg13119853	1,26	1,15	1,38	0.07	17	48939676	*TOB1*
cg15334909	1,22	1,13	1,32	0.07	2	145253398	*ZEB2*
cg07398839	0,69	0,59	0,80	0.07	15	101661834	
cg22240312	1,23	1,13	1,33	0.07	21	36430147	
cg22571060	1,22	1,13	1,32	0.07	5	143301646	
cg23512231	1,23	1,13	1,34	0.07	17	61538215	
cg13708055	1,27	1,16	1,40	0.07	15	86123699	*AKAP13*
cg05367497	1,27	1,15	1,39	0.07	7	18216902	*HDAC9*
cg04436634	1,35	1,20	1,52	0.07	1	158575773	*OR10Z1*
cg10048578	1,20	1,11	1,28	0.07	10	104533174	*WBP1L*
cg14956193	1,37	1,21	1,56	0.07	10	1058092	*GTPBP4*
cg21706867	0,74	0,65	0,83	0.07	5	54837161	
cg02783121	1,24	1,13	1,36	0.07	10	85954092	*CDHR1*
cg12011479	1,27	1,15	1,39	0.07	19	51873045	*CLDND2*
cg16257983	1,26	1,15	1,38	0.07	10	90750218	*FAS*
cg09256836	1,24	1,14	1,36	0.07	2	48036786	*FBXO11*
cg23581913	1,25	1,14	1,37	0.07	16	23516291	*GGA2*
cg03557533	1,43	1,24	1,67	0.07	3	24197658	*LOC101927854*
cg00287915	1,35	1,20	1,53	0.07	5	1503595	*LPCAT1*
cg04210225	1,43	1,24	1,66	0.07	17	43351121	*MAP3K14*
cg22968167	1,27	1,16	1,41	0.07	11	4945873	*OR51G1*
cg19951044	1,26	1,15	1,39	0.07	10	22920886	*PIP4K2A*
cg17339440	1,29	1,16	1,43	0.07	8	37743949	*RAB11FIP1*
cg01446605	1,49	1,27	1,77	0.07	19	36398148	*TYROBP*
cg12561552	1,35	1,19	1,52	0.07	1	17448350	
cg12989634	1,29	1,16	1,43	0.07	3	82344078	
cg09503310	1,26	1,15	1,39	0.07	1	1808158	*GNB1*
cg05292330	1,48	1,27	1,74	0.07	10	71929509	*SAR1A*
cg23969274	1,37	1,20	1,55	0.072	6	116851736	*FAM26D*
cg04989440	0,75	0,67	0,85	0.072	6	125684580	
cg02374401	1,41	1,22	1,62	0.072	16	15698882	*KIAA0430*
cg03841784	1,36	1,20	1,54	0.073	12	1890922	*ADIPOR2*
cg23237364	1,37	1,20	1,55	0.073	19	51308370	*C19orf48*
cg23206761	1,23	1,13	1,34	0.073	1	28210796	*C1orf38*
cg06385226	1,26	1,14	1,38	0.073	20	62539841	*DNAJC5*
cg12492850	1,38	1,21	1,58	0.073	11	6257262	*FAM160A2*
cg14039999	0,78	0,70	0,86	0.073	3	71042466	*FOXP1*
cg09710934	1,16	1,09	1,24	0.073	20	58202291	*LOC100506384*
cg08553585	1,66	1,34	2,04	0.073	1	234791553	*LOC101927787*
cg07389810	1,39	1,21	1,59	0.073	10	45800337	*OR13A1*
cg04376747	1,24	1,13	1,37	0.073	19	36120543	*RBM42*
cg22328837	1,24	1,13	1,36	0.073	6	45422120	*RUNX2*
cg24708204	1,27	1,15	1,40	0.073	8	67697201	*SGK3*
cg06224176	0,74	0,65	0,84	0.073	7	140051901	*SLC37A3*
cg10832076	1,19	1,11	1,28	0.073	12	21418929	*SLCO1A2*
cg01607516	1,31	1,17	1,46	0.073	8	103289159	*UBR5*
cg01445689	0,71	0,62	0,82	0.073	7	158703106	*WDR60*
cg01297494	1,39	1,21	1,58	0.073	10	25264136	
cg01703966	1,24	1,13	1,35	0.073	7	143207845	
cg01835527	1,27	1,15	1,39	0.073	5	148214451	
cg03668602	1,35	1,19	1,52	0.073	3	138573287	

cg21399981	1,41	1,22	1,64	0.073	1	246739638	*CNST*
cg19577080	1,21	1,12	1,31	0.073	4	143470771	*INPP4B*
cg10186605	1,28	1,16	1,42	0.073	17	29667326	*NF1*
cg03715919	1,27	1,15	1,40	0.075	22	41605644	*L3MBTL2*
cg10417386	1,20	1,11	1,29	0.075	17	8772067	*PIK3R6*
cg13340126	0,65	0,54	0,78	0.075	5	502034	*SLC9A3*
cg20792206	1,21	1,12	1,31	0.075	15	66791435	*SNAPC5*
cg06695386	1,41	1,22	1,62	0.075	5	122269911	*SNX24*
cg19862930	0,75	0,66	0,84	0.075	4	173979501	
cg14701320	1,26	1,14	1,38	0.076	6	109037239	
cg23000950	1,30	1,16	1,45	0.076	15	48785738	*FBN1*
cg04819337	1,25	1,13	1,37	0.076	21	44982367	*HSF2BP*
cg09086539	0,71	0,61	0,82	0.076	9	89563369	*LOC100506834*
cg21867373	1,51	1,27	1,78	0.077	10	102277130	*SEC31B*
cg23732483	1,27	1,15	1,40	0.078	3	48965611	*ARIH2*
cg24430106	1,54	1,28	1,85	0.078	7	30699192	*CRHR2*
cg09885622	1,27	1,15	1,39	0.078	5	1504991	*LPCAT1*
cg06899329	1,33	1,18	1,49	0.078	19	19627922	*NDUFA13*
cg23034197	1,28	1,16	1,42	0.078	2	205918689	*PARD3B*
cg23190089	1,36	1,19	1,54	0.078	11	2920209	*SLC22A18AS*
cg05934594	1,22	1,13	1,33	0.078	1	222086992	
cg27337277	1,39	1,21	1,59	0.078	19	44040457	
cg27572085	1,33	1,18	1,49	0.078	12	17742010	
cg05963604	1,32	1,17	1,49	0.08	3	57263543	*APPL1*
cg15509010	0,75	0,67	0,85	0.08	10	121412486	*BAG3*
cg27384894	1,17	1,09	1,25	0.08	11	27401878	*LGR4*
cg23478124	1,17	1,09	1,26	0.08	11	85776966	*PICALM*
cg24509193	1,27	1,15	1,40	0.08	16	18938093	*SMG1*
cg25598890	1,27	1,15	1,40	0.08	12	117501447	*TESC*
cg18667192	1,27	1,15	1,40	0.08	4	103359673	
cg25004725	1,34	1,18	1,52	0.082	5	150518408	*ANXA6*
cg26231267	0,68	0,57	0,80	0.082	10	12648032	*CAMK1D*
cg26365969	1,31	1,16	1,46	0.082	7	50626298	*DDC*
cg19538090	1,27	1,14	1,39	0.082	6	36323716	*ETV7*
cg11420400	1,37	1,20	1,56	0.082	4	153872746	*FHDC1*
cg11968932	1,32	1,17	1,48	0.082	7	8036626	*GLCCI1*
cg03411579	1,19	1,10	1,27	0.082	12	76552736	
cg08312765	1,36	1,19	1,55	0.082	12	68814447	
cg10790709	1,33	1,18	1,51	0.082	12	95698084	
cg17513953	1,25	1,14	1,38	0.082	9	129274228	
cg25597805	1,16	1,09	1,24	0.082	21	40114066	*LINC00114*
cg13089947	0,81	0,73	0,88	0.082	12	26277925	*BHLHE41*
cg02131870	0,74	0,66	0,84	0.082	7	101585659	*CUX1*
cg23625431	1,14	1,08	1,21	0.082	3	50268259	*GNAI2*
cg22185977	1,21	1,12	1,32	0.082	5	1518133	*LPCAT1*
cg18369115	1,25	1,13	1,38	0.082	5	88029636	*MEF2C*
cg00027619	1,55	1,28	1,87	0.082	2	46948734	*SOCS5*
cg07983930	1,29	1,16	1,44	0.082	1	212669100	
cg20204776	1,41	1,22	1,65	0.084	3	72010078	
cg11115176	0,83	0,77	0,90	0.085	10	116308539	*ABLIM1*
cg12036619	1,29	1,16	1,44	0.085	4	80929171	*ANTXR2*

cg06040872	1,44	1,23	1,71	0.085	17	34394215	*CCL18*
cg02798112	1,16	1,09	1,23	0.085	11	838100	*CD151*
cg16618104	1,26	1,13	1,39	0.085	12	104853100	*CHST11*
cg26702492	1,36	1,19	1,55	0.085	1	97849842	*DPYD*
cg04765471	1,21	1,11	1,30	0.085	10	11799636	*ECHDC3*
cg10736902	1,37	1,20	1,57	0.085	1	184836216	*FAM129A*
cg15199561	1,45	1,23	1,71	0.085	6	119359131	*FAM184A*
cg15119324	1,37	1,19	1,56	0.085	3	171864416	*FNDC3B*
cg20585208	1,22	1,12	1,33	0.085	21	34658768	*IL10RB*
cg17238308	1,43	1,23	1,68	0.085	15	66859511	*LCTL*
cg17720338	1,27	1,14	1,40	0.085	1	236697861	*LGALS8*
cg23542477	0,72	0,62	0,84	0.085	17	70471607	*LINC00673*
cg24811432	1,38	1,20	1,58	0.085	18	42088538	*LINC01478*
cg12793639	1,18	1,10	1,27	0.085	6	134861684	*LOC101928304*
cg00684040	1,37	1,20	1,57	0.085	5	1503632	*LPCAT1*
cg12692975	1,34	1,18	1,53	0.085	3	188475154	*LPP*
cg23331765	1,18	1,10	1,27	0.085	8	120427313	*NOV*
cg21304769	1,25	1,13	1,38	0.085	11	62563054	*NXF1*
cg26425521	1,30	1,16	1,46	0.085	7	143700939	*OR6B1*
cg12510055	0,70	0,60	0,82	0.085	20	58296411	*PHACTR3*
cg00005734	1,28	1,15	1,42	0.085	7	39662048	*RALA*
cg03718662	1,28	1,15	1,42	0.085	1	178244359	*RASAL2*
cg01375651	1,27	1,15	1,41	0.085	3	79026069	*ROBO1*
cg04442215	1,34	1,17	1,52	0.085	2	230934226	*SLC16A14*
cg20259557	1,33	1,17	1,51	0.085	8	145643626	*SLC39A4*
cg10242428	1,32	1,17	1,48	0.085	1	179286897	*SOAT1*
cg24915004	0,83	0,77	0,90	0.085	15	43001029	*STARD9*
cg09315614	1,23	1,13	1,35	0.085	13	24154719	*TNFRSF19*
cg04916103	1,25	1,13	1,38	0.085	17	4270032	*UBE2G1*
cg15359122	1,30	1,16	1,46	0.085	11	113958057	*ZBTB16*
cg02878102	1,30	1,16	1,46	0.085	19	53395635	*ZNF320*
cg00484711	0,78	0,70	0,86	0.085	2	42331922	
cg01173324	1,20	1,11	1,29	0.085	8	23600672	
cg02235628	1,32	1,16	1,48	0.085	2	208151071	
cg03172058	1,29	1,16	1,45	0.085	1	235002220	
cg05005332	1,34	1,18	1,53	0.085	9	98979142	
cg05755378	1,25	1,13	1,37	0.085	17	57496397	
cg06182994	1,28	1,15	1,42	0.085	17	34225507	
cg06339780	1,18	1,10	1,27	0.085	3	5101744	
cg06460076	1,35	1,19	1,54	0.085	11	45711402	
cg07088285	1,35	1,18	1,53	0.085	1	172498114	
cg08894366	0,86	0,81	0,91	0.085	14	91305695	
cg10151506	1,19	1,10	1,28	0.085	10	101361053	
cg10701640	0,69	0,59	0,81	0.085	17	43249399	
cg10972552	1,19	1,10	1,27	0.085	20	40490942	
cg11522179	1,28	1,16	1,43	0.085	17	21176463	
cg12683236	1,35	1,18	1,54	0.085	17	14262826	
cg14241836	1,22	1,12	1,33	0.085	7	48124921	
cg16558821	1,41	1,21	1,64	0.085	1	157832252	
cg24228311	1,34	1,18	1,52	0.085	6	16800150	
cg26922585	1,25	1,13	1,39	0.086	7	30960956	*AQP1*

cg10956275	1,29	1,16	1,44	0.086	14	75134591	*AREL1*
cg20447920	0,82	0,75	0,90	0.086	6	131555431	*AKAP7*
cg02795305	1,21	1,12	1,32	0.086	2	102481591	*MAP4K4*
cg21134737	1,21	1,11	1,31	0.087	11	2871400	
cg19700872	1,25	1,13	1,37	0.087	5	145895766	*GPR151*
cg03351159	0,78	0,70	0,87	0.088	2	242288963	*SEPT2*
cg01996125	1,20	1,10	1,29	0.088	12	52371703	*ACVR1B*
cg16760840	1,44	1,23	1,71	0.088	15	41525835	*CHP1*
cg11492856	0,77	0,68	0,86	0.088	3	15246587	*DVWA*
cg07387634	0,78	0,70	0,87	0.088	1	95006322	*F3*
cg10387956	1,39	1,21	1,61	0.088	15	72646210	*HEXA*
cg06909469	1,21	1,12	1,33	0.088	2	96990800	*ITPRIPL1*
cg07616376	1,32	1,16	1,49	0.088	18	2980515	*LPIN2*
cg04851652	1,39	1,20	1,60	0.088	7	130798757	*MKLN1*
cg19238804	0,88	0,82	0,93	0.088	17	57440226	*YPEL2*
cg02710534	0,71	0,61	0,83	0.088	5	141225619	
cg05173246	1,25	1,13	1,38	0.088	1	39042136	
cg09275783	1,35	1,18	1,53	0.088	10	77195235	
cg18175824	1,17	1,09	1,26	0.088	2	28586750	
cg20579926	1,44	1,22	1,69	0.088	11	118305240	
cg18484200	1,31	1,16	1,48	0.088	20	37213976	*ADIG*
cg24315042	1,16	1,09	1,24	0.088	12	50344303	*AQP2*
cg15942206	1,23	1,13	1,35	0.088	8	142182536	*DENND3*
cg01280180	1,19	1,10	1,28	0.088	3	138047362	*TXNDC6*
cg25099233	0,65	0,54	0,78	0.088	22	42394635	*WBP2NL*
cg20110638	1,24	1,13	1,37	0.088	3	10536903	*ATP2B2*
cg04579608	0,81	0,74	0,89	0.09	7	129410435	*MIR182*
cg00497905	0,79	0,71	0,88	0.09	11	76903183	*MYO7A*
cg00837783	1,27	1,14	1,41	0.09	2	210335358	*MAP2*
cg06487044	1,27	1,14	1,40	0.09	3	97952184	
cg06794543	0,76	0,67	0,86	0.09	15	42749748	*ZFP106*
cg15514521	1,27	1,14	1,40	0.09	11	85854937	
cg20739037	1,19	1,10	1,28	0.09	6	12473194	
cg26012092	1,25	1,13	1,38	0.09	5	179040212	
cg19802564	1,21	1,12	1,33	0.091	17	61561470	*ACE*
cg10207745	1,17	1,09	1,26	0.091	12	13529963	*C12orf36*
cg22686240	1,39	1,21	1,62	0.091	4	15375625	*C1QTNF7*
cg07301105	1,24	1,13	1,36	0.091	2	232379665	*C2orf52*
cg06740995	1,26	1,13	1,39	0.091	1	223893441	*CAPN2*
cg21921367	0,72	0,62	0,83	0.091	6	24329376	*DCDC2*
cg13062960	1,27	1,14	1,41	0.091	10	50345389	*FAM170B-AS1*
cg17407652	0,78	0,70	0,88	0.091	19	34810919	*KIAA0355*
cg19513654	1,17	1,09	1,27	0.091	4	139314416	*LINC00499*
cg01298350	1,27	1,14	1,41	0.091	5	90607970	*LUCAT1*
cg21311570	1,30	1,16	1,46	0.091	7	79087945	*MAGI2-AS3*
cg16566943	0,66	0,55	0,80	0.091	3	195530091	*MUC4*
cg14226212	1,36	1,18	1,56	0.091	9	113530240	*MUSK*
cg02465264	1,44	1,23	1,71	0.091	7	11169878	*PHF14*
cg01376665	1,23	1,12	1,35	0.091	15	40641810	*PHGR1*
cg06767389	0,88	0,82	0,93	0.091	7	157636458	*PTPRN2*
cg05456909	0,69	0,59	0,81	0.091	3	180997185	*SOX2-OT*

cg12128467	1,20	1,11	1,30	0.091	9	124116096	*STOM*
cg22889925	1,29	1,16	1,45	0.091	3	189496626	*TP63*
cg02378074	1,34	1,18	1,53	0.091	22	46734712	*TRMU*
cg01036090	1,57	1,28	1,91	0.091	12	53104746	
cg01588399	1,16	1,09	1,25	0.091	20	48364470	
cg02475902	1,18	1,09	1,27	0.091	3	195542553	
cg09351085	1,21	1,11	1,32	0.091	8	101473916	
cg12799049	1,32	1,16	1,49	0.091	1	197811487	
cg14405435	1,17	1,09	1,26	0.091	14	100500224	
cg14849880	1,22	1,12	1,34	0.091	22	46992206	
cg18758900	1,33	1,17	1,51	0.091	12	66119400	
cg20545694	1,23	1,12	1,35	0.091	8	48910778	
cg20697010	1,16	1,08	1,23	0.091	15	31683664	
cg22324068	0,81	0,74	0,89	0.091	12	67103229	
cg18458509	1,23	1,12	1,35	0.091	11	2920189	*SLC22A18AS*
cg11309217	1,51	1,25	1,80	0.092	2	216299448	*FN1*
cg07124719	1,30	1,16	1,46	0.092	4	80748552	*GDEP*
cg25348163	1,35	1,18	1,54	0.092	9	80602527	*GNAQ*
cg26057482	1,27	1,15	1,42	0.092	5	149429924	*HMGXB3*
cg01072003	1,28	1,15	1,43	0.092	6	135828046	*LINC00271*
cg17245846	1,18	1,09	1,27	0.092	12	107174001	*RIC8B*
cg23970338	1,35	1,18	1,55	0.092	12	58176483	*TSFM*
cg06892210	1,37	1,19	1,58	0.092	9	124587291	*TTLL11*
cg16541828	0,81	0,74	0,90	0.092	13	45465750	
cg22552530	1,15	1,08	1,22	0.092	5	149106296	
cg25106497	0,81	0,74	0,89	0.092	21	47160213	
cg22022320	1,47	1,24	1,75	0.092	9	32426874	*ACO1*
cg08906056	0,76	0,67	0,86	0.092	3	112214284	*BTLA*
cg08522340	1,25	1,13	1,38	0.092	11	88068493	*CTSC*
cg09906224	1,22	1,12	1,34	0.092	5	141995186	*FGF1*
cg09385130	1,23	1,13	1,36	0.092	8	125212572	*LOC101927588*
cg23985372	1,39	1,20	1,61	0.092	2	33437478	*LTBP1*
cg21060420	1,25	1,13	1,38	0.092	5	76520875	*PDE8B*
cg17407342	1,16	1,09	1,25	0.092	1	12015416	*PLOD1*
cg17233559	1,48	1,24	1,77	0.092	14	74352133	*PTGR2*
cg14889768	0,75	0,66	0,86	0.092	6	146864529	*RAB32*
cg14906565	1,33	1,16	1,51	0.092	12	49463982	*RHEBL1*
cg14485021	1,29	1,15	1,44	0.092	6	7135522	*RREB1*
cg07045119	1,30	1,16	1,46	0.092	20	19194540	*SLC24A3*
cg14646451	1,21	1,11	1,32	0.092	8	121792636	*SNTB1*
cg19347325	0,77	0,69	0,87	0.092	2	231370209	*SP100*
cg08839913	1,51	1,25	1,80	0.092	15	44955982	*SPG11*
cg23227881	1,32	1,16	1,49	0.092	16	79139758	*WWOX*
cg03647079	1,23	1,13	1,36	0.092	9	96975897	
cg04128967	1,38	1,19	1,59	0.092	14	70046847	
cg05077452	1,25	1,13	1,38	0.092	4	53614727	
cg07278844	2,22	1,55	3,18	0.092	6	109035874	
cg12372041	1,26	1,13	1,39	0.092	17	47465123	
cg13104775	1,37	1,19	1,58	0.092	4	170879928	
cg17609951	1,27	1,13	1,40	0.092	21	17064955	
cg17975283	1,24	1,13	1,37	0.092	10	64504116	

cg18536308	1,16	1,08	1,23	0.092	17	43412082	
cg19853852	1,28	1,15	1,43	0.092	1	226548273	
cg23683800	1,33	1,16	1,51	0.092	10	115086103	
cg08870682	1,51	1,25	1,82	0.092	6	123124606	*SMPDL3A*
cg03042890	1,33	1,16	1,51	0.092	14	22279274	
cg15776495	1,28	1,14	1,43	0.092	16	89428646	*ANKRD11*
cg14343337	1,27	1,14	1,41	0.092	19	33161082	*ANKRD27*
cg25202593	0,83	0,76	0,90	0.092	3	14729104	*C3orf20*
cg08394012	1,33	1,17	1,52	0.092	7	132749681	*CHCHD3*
cg21793826	1,31	1,16	1,47	0.092	16	55562323	*LPCAT2*
cg14642787	1,18	1,09	1,27	0.092	17	17882518	*LRRC48*
cg17599983	0,80	0,72	0,88	0.092	17	16216940	*PIGL*
cg06716519	1,25	1,13	1,39	0.092	1	214168416	*PROX1*
cg01740552	1,40	1,21	1,64	0.092	10	89714113	*PTEN*
cg01116484	1,21	1,11	1,32	0.092	11	116943299	*SIK3*
cg01569173	1,29	1,15	1,44	0.092	8	98288910	*TSPYL5*
cg02277605	1,24	1,13	1,37	0.092	2	201167826	
cg07136652	1,24	1,13	1,36	0.092	15	39627235	
cg08884974	1,27	1,14	1,41	0.092	15	31505024	
cg10580537	1,29	1,16	1,45	0.092	12	31390583	
cg14002425	1,28	1,15	1,43	0.092	9	73148415	
cg20339868	1,27	1,14	1,41	0.092	2	7913670	
cg02072495	1,21	1,11	1,32	0.094	15	60689285	*ANXA2*
cg15893925	1,25	1,13	1,38	0.094	6	134758804	*LINC01010*
cg14884379	1,27	1,13	1,40	0.094	18	61559440	*SERPINB2*
cg06612119	1,21	1,11	1,31	0.094	12	56404209	
cg20033221	1,26	1,13	1,39	0.094	15	101743422	*CHSY1*
cg11552868	0,87	0,81	0,93	0.094	11	128424176	*ETS1*
cg06848181	1,36	1,18	1,57	0.094	15	63687276	*LOC102723344*
cg19937039	1,24	1,13	1,37	0.094	14	94759767	*SERPINA10*
cg00042059	1,39	1,19	1,60	0.094	2	55087588	*EML6*
cg15197144	1,42	1,21	1,67	0.094	19	3521010	*FZR1*
cg19853760	1,14	1,07	1,21	0.094	22	38071677	*LGALS1*
cg13810556	1,23	1,12	1,35	0.094	2	217363253	*RPL37A*
cg04682911	0,81	0,73	0,89	0.094	8	59971099	*TOX*
cg20519597	1,34	1,17	1,53	0.094	11	33156606	*CSTF3*
cg03909307	1,31	1,16	1,48	0.094	9	93341167	*LINC01501*
cg19688761	1,28	1,14	1,43	0.094	10	129785464	*PTPRE*
cg21565368	1,48	1,24	1,79	0.094	11	85437579	*SYTL2*
cg18542882	1,17	1,09	1,27	0.094	19	3008169	*TLE2*
cg09463453	1,17	1,09	1,27	0.095	10	79633462	*DLG5*
cg18431127	1,16	1,09	1,26	0.095	15	43513563	*EPB42*
cg26421610	1,35	1,17	1,54	0.095	11	118958280	*HMBS*
cg07913379	1,23	1,12	1,36	0.095	1	200325145	*LINC00862*
cg17788613	1,27	1,14	1,41	0.095	13	67432477	*PCDH9-AS2*
cg15323433	1,27	1,14	1,41	0.095	14	102344974	*PPP2R5C*
cg12736613	1,31	1,16	1,48	0.095	7	151501146	*PRKAG2*
cg12048225	0,81	0,74	0,90	0.095	6	32808669	*PSMB8*
cg17398932	1,37	1,18	1,57	0.095	13	41362292	*SLC25A15*
cg14255094	0,84	0,77	0,91	0.095	7	127730344	*SND1*
cg02737765	1,40	1,21	1,64	0.095	5	146725861	*STK32A*

cg10402121	1,31	1,16	1,48	0.095	10	126487101	
cg11283005	1,42	1,21	1,67	0.095	7	37529545	
cg24698838	1,22	1,12	1,35	0.095	2	129199624	
cg26363753	1,18	1,09	1,27	0.095	3	177913761	
cg27296554	1,33	1,16	1,51	0.095	11	133692122	
cg27661078	1,55	1,27	1,89	0.095	6	14724942	
cg03228907	1,35	1,17	1,54	0.095	18	19032045	*GREB1L*
cg16220260	1,39	1,20	1,62	0.095	1	204386605	
cg22177553	1,27	1,13	1,40	0.095	11	60602772	
cg17092005	1,29	1,15	1,45	0.1	11	134258492	*B3GAT1*

Supplemental Table S2. Top 10 DMPs in girls

CpG site	2^logFC	CI left	CI right	p.value	q. Value	CHR	Position	Gene
cg14111928	1,24	1,14	1,35	9,58E-06	1	10	76602391	*MYST4*
cg15817199	1,30	1,17	1,45	1,42E-05	1	14	22938722	
cg15199561	1,37	1,21	1,56	1,51E-05	1	6	119359131	*FAM184A*
cg02053587	0,67	0,57	0,79	1,76E-05	1	17	3599516	*P2RX5*
cg10608169	1,48	1,26	1,75	2,67E-05	1	9	37964630	*SHB*
cg12259655	0,79	0,71	0,87	2,96E-05	1	16	29743655	
cg23062357	1,31	1,16	1,47	4,19E-05	1	14	22938675	
cg12569118	0,50	0,37	0,67	4,34E-05	1	11	74394623	
cg21750602	1,36	1,19	1,56	4,90E-05	1	10	133748026	*PPP2R2D*
cg00867453	0,75	0,66	0,85	5,06E-05	1	9	88555863	*NAA35*

Supplemental Table S3. Top 10 DMPs in boys

CpG site	2^logFC	CI left	CI right	p.value	q. Value	CHR	Position	Gene
cg16813892	1,44	1,26	1,64	2,84E-06	1	1	93544665	*MTF2*
cg22262325	0,65	0,55	0,77	1,40E-05	1	8	141849588	*PTK2*
cg03557533	1,30	1,17	1,45	2,38E-05	1	3	24197658	*LOC101927854*
cg12433790	0,66	0,54	0,79	7,09E-05	1	1	19274958	*IFFO2*
cg26709950	1,37	1,19	1,58	7,31E-05	1	16	66959235	*RRAD*
cg18583094	1,37	1,19	1,57	7,32E-05	1	11	122752205	*C11orf63*
cg01176363	1,33	1,17	1,51	7,53E-05	1	20	62369445	*LIME1*
cg13725340	1,35	1,18	1,56	0,0001023	1	9	135432492	*C9orf171*
cg24201362	1,37	1,18	1,58	0,0001030	1	6	467681	
cg11868921	1,34	1,17	1,53	0,0001045	1	6	154812487	*CNKSR3*
